# Cover

**DOI:** 10.4269/ajtmh.1124scover

**Published:** 2025-04-01

## Abstract

A health worker conducts a COVID-19 rapid test in Suriname. *Photo Credit: Timothy Henny/Slingshot*

Training for COVID-19 rapid testing in Suriname. *Photo Credit: Timothy Henny/Slingshot*

A health worker in Suriname shows her son how a COVID-19 rapid test works. *Photo Credit: Timothy Henny/Slingshot*

COVID-19 rapid testing on a patient in Suriname. *Photo Credit: Timothy Henny/Slingshot*

